# Craniofacial morphology in down syndrome: a systematic review and meta-analysis

**DOI:** 10.1038/s41598-020-76984-5

**Published:** 2020-11-16

**Authors:** Ascensión Vicente, Luis-Alberto Bravo-González, Ana López-Romero, Clara Serna Muñoz, Julio Sánchez-Meca

**Affiliations:** 1 Department of Orthodontics, Faculty of Medicine, University of Murcia, Murcia, Spain; 2 Department of Integral Pediatric Dentistry, Hospital Morales Meseguer, University of Murcia, 2ª Planta, C/Marqués de los Vélez s/n, 30008 Murcia, Murcia Spain; 3 Department of Basic Psychology and Methodology, University of Murcia, Murcia, Spain

**Keywords:** Dentistry, Orthodontics, Craniofacial orthodontics

## Abstract

The aim of this study was to evaluate the craniofacial cephalometric characteristics of individuals with Down syndrome (DS), comparing them with healthy subjects. An electronic search was made in Pubmed, Embase, Lilacs, Scopus, Medline and Web of Science without imposing limitations on publication date or language. Studies were selecting following the Preferred Reporting Items for Systematic Reviews and Meta-analyses (PRISMA) statement. The PECO acronym was applied as follows: P (population), individuals with DS; E, (exposition) diagnosis of DS; C (comparison), individuals without DS; O (outcomes) craniofacial characteristics based on cephalometric measurements. Independent reviewers performed data extraction and assessed the methodological quality of the articles using the Newcastle–Ottawa Quality-Assessment-scale. Seven case–control studies were included in meta-analysis. Given the variability of the cephalometric measurements used, only those that had been reported in at least three or more works could be included. Anterior cranial base length (SN), posterior cranial base length (SBa), total cranial base length (BaN), effective length of the maxilla (CoA), sagittal relationship between subspinale and supramentale (ANB), anterior facial height (NMe), and posterior facial height (SGo) values were significantly lower in the DS population than among control subjects. No significant differences were found in sagittal position of subspinale relative to cranial base (SNA) and sagittal position of supramentale relative to cranial base (SNB). Summarizing, individuals with DS present a shorter and flatter cranial base than the general population, an upper jaw of reduced sagittal dimension, as well as a tendency toward prognatic profile, with the medium third of the face flattened and a reduced anterior and posterior facial heights.

## Introduction

In 1866, John Langdon Haydon Down first described the characteristics presented by individuals with DS^1^. This syndrome has a prevalence that ranges between 1 per 800–1200 live births^2^. It is caused by partial or complete triplication of chromosome 21 and is the most common genetic developmental disorder^3^. Among possible risk factors for these cytogenetic disorders is the mother’s age, whereby incidence reaches one in every 12 births in women aged over 49 years^4^. But there is some controversy regarding paternal age as a risk factor, so that some researchers find no relation^5,6^, while others suggest that there is a moderate increase in risk when fathers are aged over 40 years^7,8^.

Individuals with DS may present multiple health problems, including varying degrees of intellectual capacity, as well as delayed speech and learning development. In early adulthood, DS individuals are at considerable risk of developing dementia and Alzheimer disease^3^. In addition, ophthalmological problems, hypothyroidism, epilepsy, obstructive sleep apnea and transient myeloproliferative disorders are more common in this group^2^.

Regarding dental status, DS patients often present delays and disorders in the eruption sequence of both deciduous^9,10^ and permanent dentition^11^. Dental agenesis is common and is recognized as one of the phenotypic characteristics of DS. The prevalence of agenesis (excluding third molars) is higher than among healthy individuals, present in approximately 54.6–58.5% of subjects with DS^12^. Microdontia^13^ and taurodontism^14^ are also fairly common. Regarding malocclusions, a higher prevalence of Angle Class III malocclusion, posterior cross bite, and anterior open bite has also been observed among DS patients^15^.

The craniofacial characteristics of individuals with DS have been described in various studies, but to date no systematic review of published research has been conducted. So the objective of the present work was to perform meta-analysis to evaluate the craniofacial cephalometric characteristics of individuals with DS in comparison with healthy populations, allowing us to synthesize available scientific information in the field for the first time, increasing the validity of the conclusion of quality primary studies. The review’s null hypothesis was that there are no differences in cephalometric characteristics derived from the analysis of lateral teleradiographs of the head between individuals with DS and the general population.

## Materials and methods

### Protocol and registration

This systematic review followed *Preferred Reporting Items for Systematic Reviews and Meta-Analyses* (PRISMA) guidelines^16^ and was registered in the PROSPERO database (International Prospective Register of Systematic Reviews) (Reg. No. CRD42018117175) at the University of York (U.K.).

### Selection criteria

The population, exposition, comparison, outcome (PECO)^17^ acronym was applied to answer the following question: “Are the craniofacial characteristics of individuals with DS different from those of the general population?”. PECO was applied as follows: P, individuals with DS; E, clinical or genetic diagnosis of DS; C, individuals without DS; O, craniofacial characteristics of DS evaluated by means of lateral cephalometric measurements derived from the analysis of lateral teleradiographs of the head.

Inclusion criteria were: case–control, cross-sectional and cohort studies describing cephalometric measurements of subjects with DS, compared with healthy control subjects. Animal studies, clinical case reports, pilot studies, bibliographic reviews, systematic reviews, and chapters of books were excluded.

### Search strategy

A thorough search was conducted without imposing any limitations on publication date or language in the following databases: Pubmed, Embase, Lilacs, Scopus, Medline, and Web of Science. The search was completed on 27th May 2019. The following search terms were used: “Trisomy 21”; “Down syndrome”; “Mongolism”; and “Trisomy G” combined (using Boolean operator “AND”) with “craniofacial characteristics”; “craniofacial features”; “craniofacial development”; “craniofacial growth”; “craniofacial morphology”; “craniofacial cephalometric”; and “dento-skeletal”.

The electronic search was complemented by a manual search in specialist journals: The Angle Orthodontics, American Journal of Orthodontics and Dentofacial Orthopedics, and the European Journal of Orthodontics.

### Article selection

**Figure 1:**
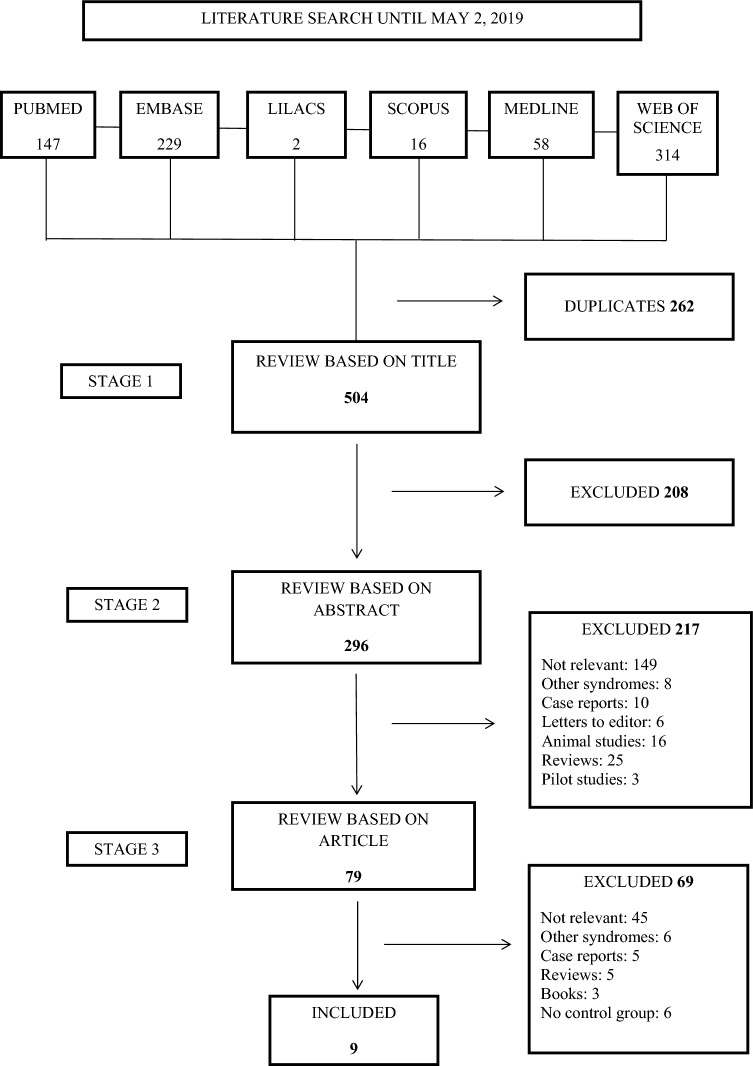
Flow diagram of the search strategy.

The selection of articles was made in three stages as shown in Fig. 1. Two researchers (A.V and A.L) carried out the selection process independently; any disagreement over the results was resolved by consensus. When agreement could not be reached, a third assessor was consulted (C.S.).

A manual search was also conducted among the bibliographic references of the articles identified in the initial search.

The degree of agreement between reviewers was assessed by means of Cohen’s kappa coefficient (κ).

### Methodological quality assessment

The methodological quality of the articles selected was assessed using the Newcastle–Ottawa Quality Assessment scale (NOS)^18^ by two examiners working together (A.V and A.L) and a third examiner (C.S) working independently. The degree of agreement between the examiners was analyzed with Cohen’s kappa coefficient (κ).

The NOS gives a maximum score of nine; articles scoring seven or above are considered of good quality. Assessment is divided into three areas: selection, comparability and exposure. The selection criterion consisted of four items: adequate definition of cases, representativity of the cases, selection of controls, and definition of controls. Comparability consists of the comparability of cases and controls derived from study design, considering factors such as age or ethnicity. The last part is exposure, which is divided into three items: evaluation of exposure, equality of methods for cases and controls, and dropout rate.

There is a maximum score of one for each item, with the exception of comparability, which can award a score of two.

### Data extraction

**Figure 2:**
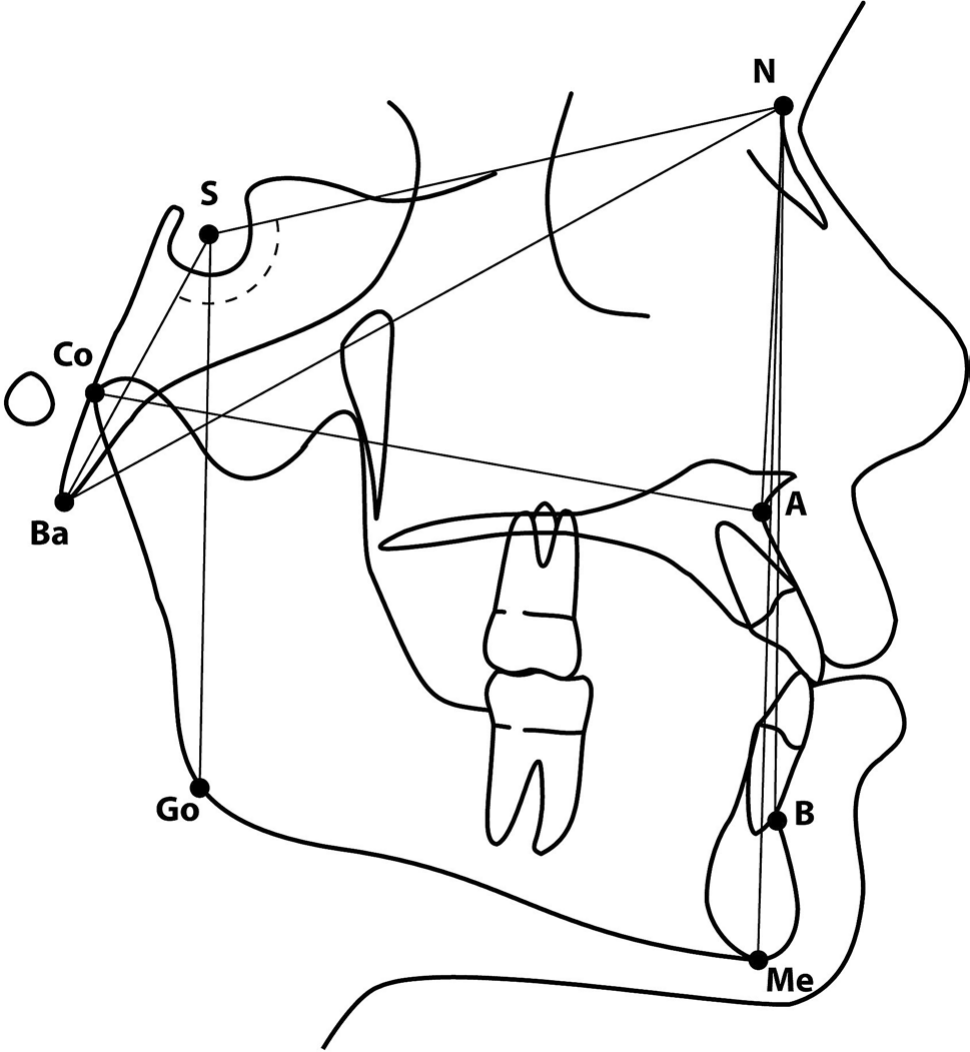
Cephalometric landmarks (*S* Sella, *N* Nasion, *Ba* Basion, *A* A point, *Co* Condilion, *B* B point, *Me* Menton, *Go* Gonion), Cranial base measurements (SN length, SBa length, BaN length, SNBa angle), Maxilar measurements (SNA angle, CoA length), Mandibular measurements (SNB angle), Maxilo-Mandibular relation (ANB angle), Facial heights (NMe length, SGo length).

The following data were extracted from each of the articles by two researchers (A.V. and C.S): authors, year of publication and for both cases and controls sample size, ethnicity, gender, and cephalometric measurements were registered. Given the wide variety of cephalometric measurements taken in the articles, for meta-analysis it was decided to include only those measurements that were repeated in at least three articles, these being: Anterior cranial base legth (SN, mm), posterior cranial base length (SBa, mm), total cranial base length (BaN, mm), posterior cranial base inclination (SNBa, °), sagittal position of subspinale relative to cranial base (SNA, °), effective length of the maxilla (CoA, mm), sagittal position of supramentale relative to cranial base (SNB, °), relative position of maxilla and mandible to each other (ANB, °), anterior facial height (NMe, mm), posterior facial height (SGo, mm) (Fig. 2; Table 1). The mean value and standard deviation were recorded for each measurement.

**Table 1:** Angular and linear cephalometric measurements and their definitions.

Cephalometric measurements	Definitions
**Cranial base**	
S-N length, mm	The distance between Sella point and Nasion: anterior cranial base length
S-Ba length, mm	The distance between Sella and Basion: posterior cranial base length
Ba-N length, mm	The distance between Basion and Nasion: total cranial base length
S-N-Ba angle	Posterior cranial base inclination
**Maxilar**	
S-N-A angle	Angle between cranial base to subspinale (A-point): sagittal position of subspinale relative to cranial base
Co-A, mm	Length of the line drawn from Condilion to A point: effective length of the maxilla
**Mandibular**	
S-N-B angle	Angle between cranial base to supramentale (B-point): sagittal position of supramentale relative to cranial base
**Maxilo-mandibular relation**	
ANB angle	Angle between N-A and N-B: relative position of maxilla and mandible to each other
**Face heights**	
N-Me length, mm	The distance between Nation and Menton: anterior facial height
S-Go length, mm	The distance between Sella point and Gonion: posterior facial height

The reliability of the codification process between examiners was analyzed by means of Cohen’s kappa coefficient for qualitative variables and the intra-class correlation coefficient for quantitative variables.

### Effect size index

For each outcome the effect size index was the mean difference (MD) between the DS group and the control group. A negative value for the MD indicated that a mean in the DS group was lower than that of the control group, and vice versa.

### Statistical analysis

Separate meta-analyses were carried out for each outcome. As different articles varied in the cephalometric measurements taken, the results could only be subjected to meta-analysis when a mean datum was reported in at least three studies. As some evidence of heterogeneity among the individual studies was expected, a random-effects model was assumed. This model implies to weight each effect size by its inverse variance, this being the inverse of the sum of two variances: within-study variance and between-studies variance. Between-studies variance was estimated using the DerSimonian and Laird estimator. For each outcome, a pooled effect size (in terms of mean difference) was calculated, as well as the 95% confidence interval^19^. Heterogeneity of the effect sizes was checked with the *Q* statistic and *I*^2^ index. A statistically significant result for the *Q* statistic (*p* < 0.05) indicated the presence of heterogeneity. In addition, heterogeneity *I*^2^ indices around 25%, 50%, and 75% were considered to reflect low, moderate, and large heterogeneity, respectively^20^. Finally, to assess the potential influence of the two age ranges found in the studies (6–11 years old and 5–22 years old), subgroup analyses were applied with the *Q*_B_ statistic. A statistically significant result for the *Q*_B_ statistic (*p* < 0.05) provided evidence of significant differences between the average effect sizes of the age ranges. In addition, a double forest plot was constructed for each outcome in order to illustrate the potential differences among the effect sizes as a function of the age range. All statistical analyses were carried out using Comprehensive Meta-analysis 3.3 software^21^.

## Results

### Study selection

The search identified 766 articles, 147 in PubMed, 229 in Embase, 2 in Lilacs, 16 in Scopus, 58 in Medline, and 314 in Web of Science. After excluding duplicates, 504 articles remained. A further 208 were rejected on the basis of the title leaving 296 articles, of which 79 were selected after reading the abstracts. Then the full texts were read and a further 69 excluded for failure to meet the review criteria, leaving a total of 9 works (Fig. 1). The Cohen kappa coefficient of inter-examiner reliability was κ = 0.890.

The manual search in journals did not identify any additional works.

### Methodological quality assessment

The nine articles selected were assessed with the NOS^18^, obtaining a Cohen kappa coefficient of κ = 0.832 between evaluators.

Of the nine articles, seven underwent quality assessment; four obtained maximum scores of nine^22–25^, two a score of eight^26,27^, and one of seven^28^. The two articles considered of low quality^9,29^ obtained scores of six (Table 2) and they were excluded from the meta-analysis.

**Table 2:** Methodological quality for all 9 case control studies identified by the search strategy, assessed using the Newcastle–Ottawa-Scale.

References	Country	Study design	Criteria^a^									
			Selection				Comparability		Exposure			Total score
			1	2	3	4	5		6	7	8	
Tosso and Naval^26^	France	Case–control	*	*	*	*	*		*	*	*	8
Fischer-Brandies^9^	Germany	Case–control	*	*	*	*	*				*	6
Clarkson et al.^28^	Colombia	Case–control	*		*	*	*		*	*	*	7
Alió et al.^22^	Spain	Case–control	*	*	*	*	*	*	*	*	*	9
Suri et al.^23^	Canada	Case–control	*	*	*	*	*	*	*	*	*	9
Silva and Valladares-Neto^27^	Brazil	Case–control	*	*		*	*	*	*	*	*	8
Alió et al.^24^	Spain	Case–control	*	*	*	*	*	*	*	*	*	9
Korayem and Alkofide^25^	Saudi Arabia	Case–control	*	*	*	*	*	*	*	*	*	9
Allareddy et al.^29^	USA	Case–control	*	*		*			*	*	*	6

^a^(1) Adequate case definition, (2) Representativeness of the cases, (3) Selection of controls, (4) Definition of controls, (5) Comparability of Cases and Controls on the basis of the design or analysis (age y ethnicity), (6) Ascertainment of exposure, (7) Same method of ascertainment for cases and controls, (8) Non-response rate.

### Reliability of data extraction process

The degree of inter-examiner agreement was high. For all quantitative variables, except for the item “number of controls,” an intra-class correlation coefficient of one was obtained. Initially the intra-class correlation coefficient for “number of controls” was 0.77 and after finding the mistake in the number of controls between the examiners we corrected it and obtained a κ = 1. Qualitative variables all obtained values of κ = 1.

### Study characteristics

Table 3 summarizes details of the seven case–control studies included in meta-analysis. Five articles were published in English^22–25,27^, one in French^26^ and one in Spanish^28^. The earliest publication date was 1985^26^, while the most recent was published in 2014^25^. Studies were conducted in Canada^23^, France^26^, Colombia^28^, Spain^22,24^, Brazil^27^ and Saudi Arabia^25^. The smallest sample size was 14 individuals^28^, while the maximum was 60^25^. The ages of the subjects investigated ranged from 5^27^ to 22 years^25^. In five articles the individuals were Caucasian^22–24,26,27^, in one Columbian^28^, and in the other Arab^25^. All the works coincided in age range and ethnicity between case and control groups. Regarding gender, five works included individuals of both sexes^22–26^; one included only males^27^, while another one did not report the sex of the subjects^28^.

**Table 3:** Study characteristics of the 7 case–control studies included in the sistematic review and meta-analyses.

References	Nos	Partipants	Age range	Ethnicity	Sex	Cephalometric measurements (Mean ± SD)
Alió et al.^22^	9	Cases: 47	Cases: 8–18	Cases: Caucasian	Cases: 22 ♀ 25♂	SN (mm)
		Controls: 38	Controls: 8–18	Controls: Caucasian	Controls: 16 ♀ 22♂	Cases: 63.67 ± 4.04 Controls:72.26 ± 2.84
						SBa(mm)
						Cases: 42.33 ± 3.22 Controls: 46.30 ± 3.03
						NBa (mm)
						Cases: 100.9 ± 5.55 Controls: 109.6 ± 4.36
						SNBa (°)
						Cases: 143.4 ± 4.76 Controls: 133.9 ± 4.12
Clarkson et al.^28^	7	Cases: 14	Cases: 8–11	Cases: Colombian	Cases: No data	SN (mm)
		Controls: 14	Controls: 8–11	Controls: Colombian	Controls: No data	Cases:63.3 ± 3.48 Controls:69.10: ± 3.48
						SNA (°)
						Cases: 80.5 ± 2.68 Controls: 82.5 ± 2.68
						ANB (°)
						Cases:3.3 ± 2.48 Controls: 4.7 ± 2.48
						NMe (mm)
						Cases: 103.5 ± 6.99 Controls: 113.2 ± 6.99
						SGo (mm)
						Cases:65.7 ± 5.56 Controls:72.9 ± 5.56
Silva and Valladares-Neto^27^	8	Cases: 30	Cases: 6–11	Cases: Caucasian	Cases: 30♂	SN (mm)
		Controls: 30	Controls: 6–11	Controls: Caucasian	Controls: 30♂	Cases: 62.2 ± 3.78 Controls: 69.6 ± 4.22
						SBa(mm)
						Cases: 42.2 ± 3.08 Controls: 44.20 ± 2.84
						NBa (mm)
						Cases: 96.6 ± 5.28 Controls: 103.3 ± 5.86
						SNBa (°)
						Cases: 134.6 ± 4.9 Controls: 128.1 ± 2.85
						SNA (°)
						Cases: 79.9 ± 3.91 Controls: 80.9 ± 3.19
						CoA (mm)
						Cases: 75.4 ± 4.56 Controls: 84.8 ± 4.36
						SNB (°)
						Cases: 78.4 ± 4.3 Controls: 81 ± 3.14
						ANB (°)
						Cases: 1.4 ± 2.9 Controls: 3.6 ± 1.98
						NMe (mm)
						Cases: 97.7 ± 7.28 Controls: 108.3 ± 5.59
						SGo (mm)
						Cases: 62.7 ± 5.9 Controls: 66.7 ± 4.71
Suri et al.^23^	9	Cases: 25	Cases: 11–18	Cases: Caucasian	Cases: 13 ♀ 12♂	SN (mm)
		Controls: 25	Controls: 11–18	Controls: Caucasian	Controls: 13 ♀ 12♂	Cases: 64.97 ± 3.52 Controls: 75.17 ± 3.74
						SBa(mm)
						Cases: 44.46 ± 3.05 Controls: 48.40 ± 3.01
						NBa (mm)
						Cases: 103.08 ± 5.13 Controls: 112.48 ± 5.3
						SNBa (°)
						Cases: 140.31 ± 3.75 Controls: 129.92 ± 4.06
						SNA (°)
						Cases: 82.47 ± 4.34 Controls: 81.25 ± 2.87
						SNB (°)
						Cases: 82.41 ± 4.36 Controls: 78.74 ± 2.64
						ANB (°)
						Cases: 0.06 ± 2.51 Controls: 2.52 ± 1.48
						NMe (mm)
						Cases: 106.23 ± 8.04 Controls: 121.74 ± 6
						SGo (mm)
						Cases: 70.36 ± 8.88 Controls: 78.83 ± 6.45
Korayem and Alkofide^25^	9	Cases: 60	Cases: 12–22	Cases: Arab	Cases: 33 ♀ 27♂	SN (mm)
		Controls: 60	Controls: 12–22	Controls: Arab	Controls: 33 ♀ 27♂	Cases: 65.20 ± 4.4 Controls: 72.90 ± 3.6
						SBa(mm)
						Cases: 44.5 ± 3.3 Controls: 46.5 ± 3.3
						SNBa (°)
						Cases: 138.53 ± 3.83 Controls:130.23 ± 1.96
						SNA (°)
						Cases: 81.9 ± 2.4 Controls: 83.3 ± 2.5
						CoA (mm)
						Cases: 85.7 ± 5.80 Controls: 92.7 ± 3
						SNB (°)
						Cases: 81.4 ± 3 Controls: 80.4 ± 2.7
						ANB (°)
						Cases: 0.54 ± 2.6 Controls: 3.1 ± 0.9
Alió et al.^24^	9	Cases: 47	Cases: 8–18	Cases: Caucasian	Cases: 22 ♀ 25♂	SNA (°)
		Controls: 38	Controls: 8–18	Controls: Caucasian	Controls: 16 ♀ 22♂	Cases: 78.85 ± 3.44 Controls: 79.27 ± 3.18
						CoA (mm)
						Cases: 78.89 ± 5.79 Controls: 88.57 ± 4.65
Alonso Tosso and Naval^26^	8	Cases: 33	Cases: 5–19	Cases: Caucasian	Cases: 15 ♀ 18♂	SNBa (°)
		Controls: 45	Controls:5–19	Controls: Caucasian	Controls: 22 ♀ 23♂	Cases: 138.96 ± 4.13 Controls: 130.84 ± 5.56

Cephalometric characteristic of the cranial base were assessed by means of the measurements SN^22,23,25,27,28^; SBa^22,23,25,27^; NBa^22,23,27^; and SNBa^22,23,25–27^. Measurements relating to the maxilla were SNA^23–25,27,28^ and CoA^24,25,27^; and to the mandible SNB^23,25,27^. Anteroposterior relation between maxilla and mandible was determined by means of the ANB angle^23,25,27,28^; anterior face height by NMe^23,27,28^; and posterior face height by means of SGo^23,27,28^.

### Meta-analyses

As shown in Table 4, a total of five studies were found to report outcomes. With the exception of SNBa and SNB, lower mean outcomes were found among DS subjects than controls. Moreover, the DS group exhibited significantly lower mean values than the control group for outcomes SN, SBa, NBa, CoA, ANB, NMe, and SGo. Two outcomes (SNBa and SNB) obtained higher mean values for DS subjects than controls but only the difference in SNBa reached statistical significance.

**Table 4:** Results of the pooled mean difference and heterogeneity statistics for each outcome.

Outcome	*k*	MD_+_	95% CI		*Q*	*df*	*p*	*I*^2^ (%)
			MD_L_	MD_U_				
SN (mm)	5	− 8.036	− 9.224	− 6.848	8.24	4	.083	51.5
SBa (mm)	4	− 2.931	− 4.048	− 1.815	7.52	3	.057	60.1
NBa (mm)	3	− 8.336	− 9.811	− 6.860	1.92	2	.383	0
SNBa (°)	5	8.518	7.391	9.646	7.95	4	.093	49.7
SNA (°)	5	− 0.841	− 1.742	0.060	6.96	4	.138	42.5
CoA (mm)	3	− 8.546	− 10.358	− 6.734	4.71	2	.095	57.5
SNB (°)	3	0.688	− 2.286	3.661	20.30	2	< .001	90.1
ANB (°)	4	− 2.388	− 2.903	− 1.872	1.45	3	.695	0
NMe (mm)	3	− 12.052	− 15.574	− 8.530	4.51	2	.105	55.7
SGo (mm)	3	− 6.137	− 8.965	− 3.309	3.62	2	.163	44.8

*k*, number of studies. MD_+_, pooled mean difference. MD_L_ and MD_U_, lower and upper limits of the 95% confidence interval around MD_+_. *Q*, heterogeneity statistic. *DF*, degrees of freedom of the *Q* statistic. *p*, probability level of the *Q* statistic. *I*^2^, heterogeneity index.

Due to the low number of studies identified, the interpretation of *Q* statistics must be treated with caution, due to problems of statistical power. The *I*^2^ index offers a more adequate means of assessing heterogeneity among effect sizes. As shown in Table 3, moderate-to-high heterogeneity was found for all outcomes, with the exception of NBa and ANB.

**Figure 3:**
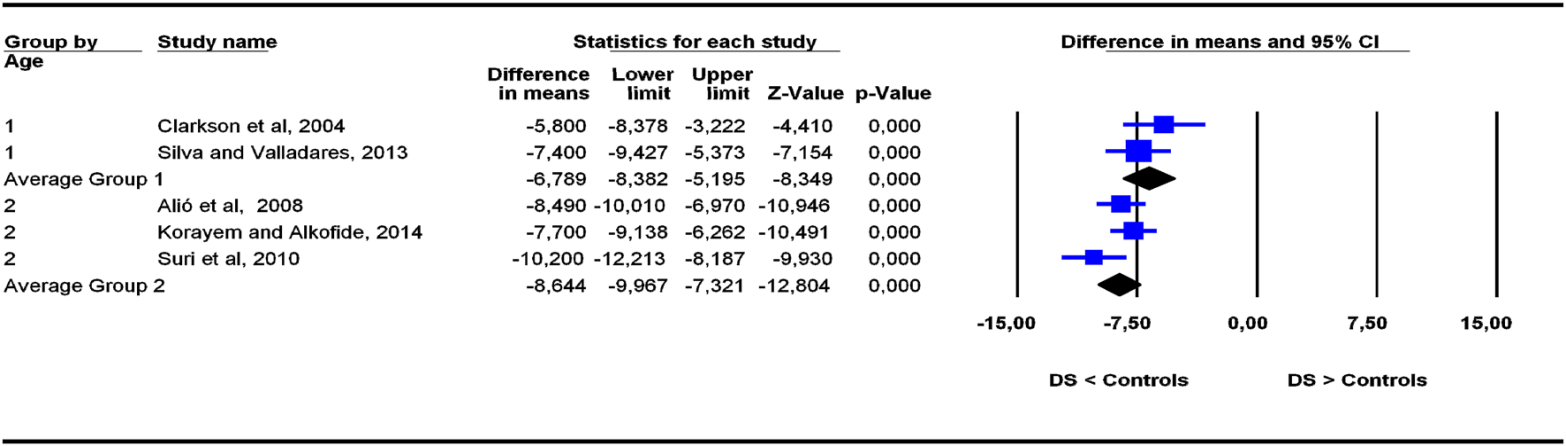
Forest plot for the outcome ‘SN (mm)’ as a function of the age group (1 = 6–11 years old), 2 = 5–22 years old).

**Figure 4:**
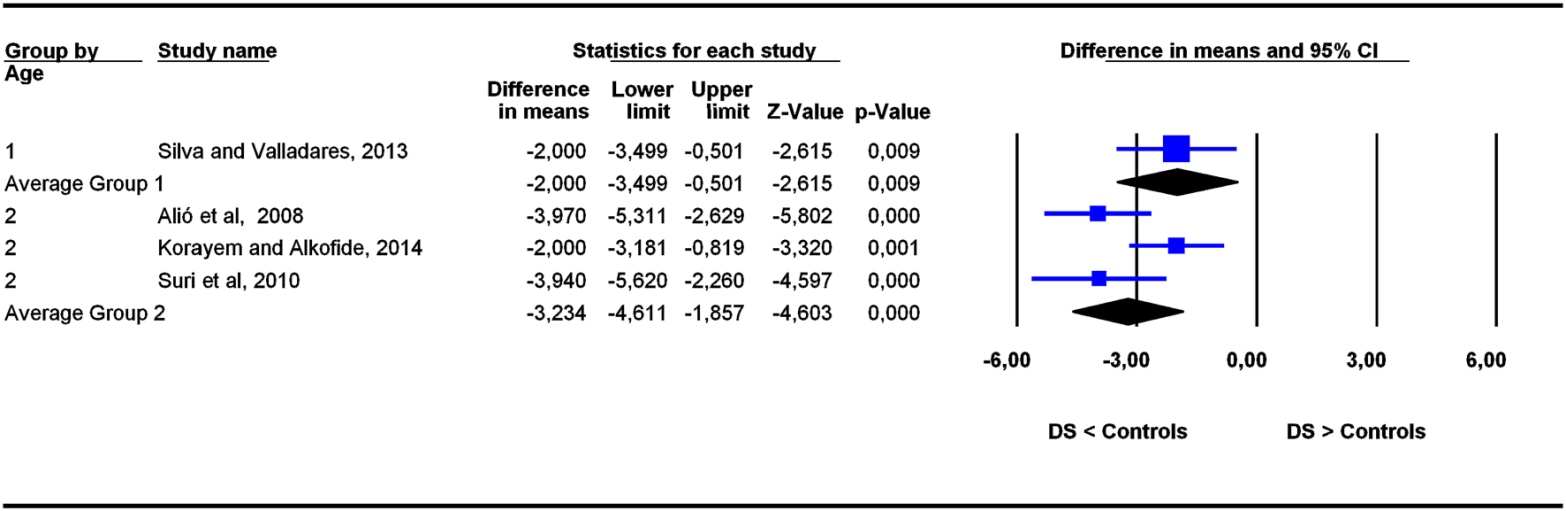
Forest plot for the outcome ‘SBa (mm)’ as a function of the age group (1 = 6–11 years old), 2 = 5–22 years old).

**Figure 5:**
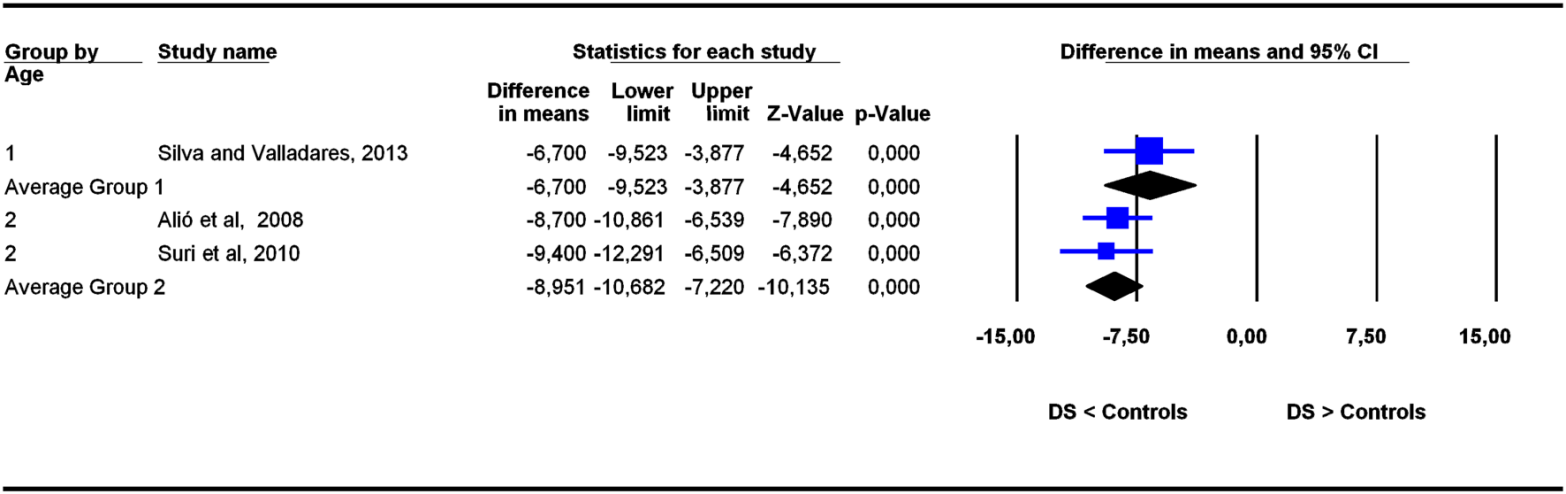
Forest plot for the outcome ‘BaN (mm)’ as a function of the age group (1 = 6–11 years old), 2 = 5–22 years old).

**Figure 6:**
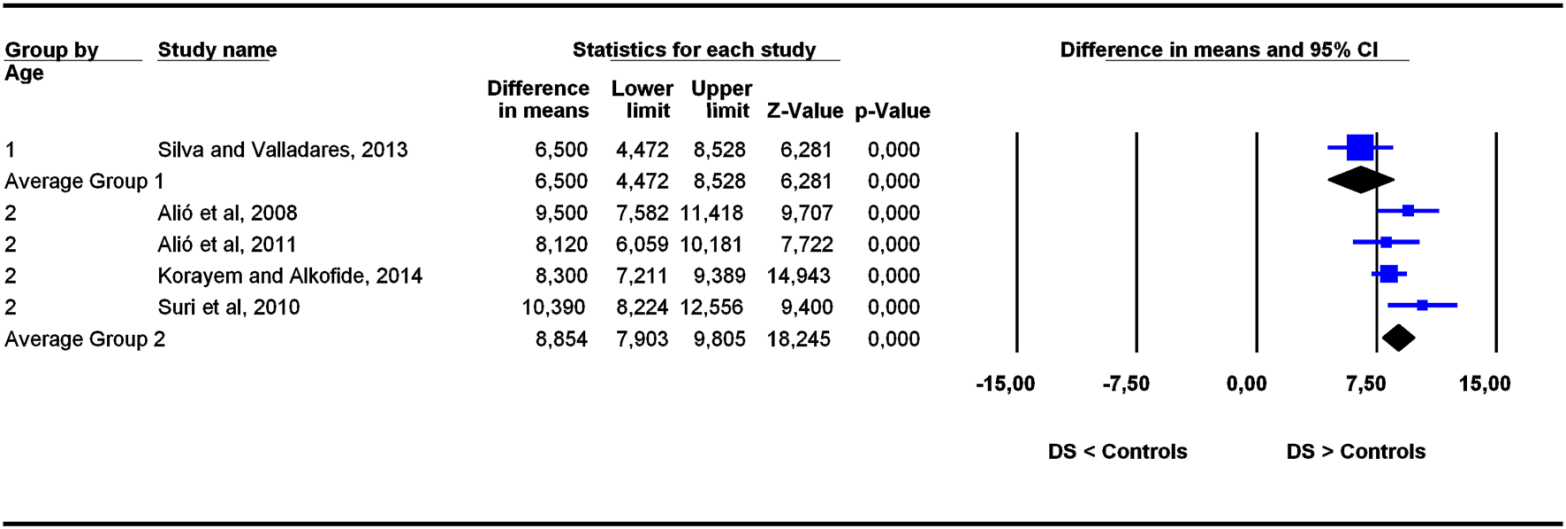
Forest plot for the outcome ‘SNBa (°)’ as a function of the age group (1 = 6–11 years old), 2 = 5–22 years old).

**Figure 7:**
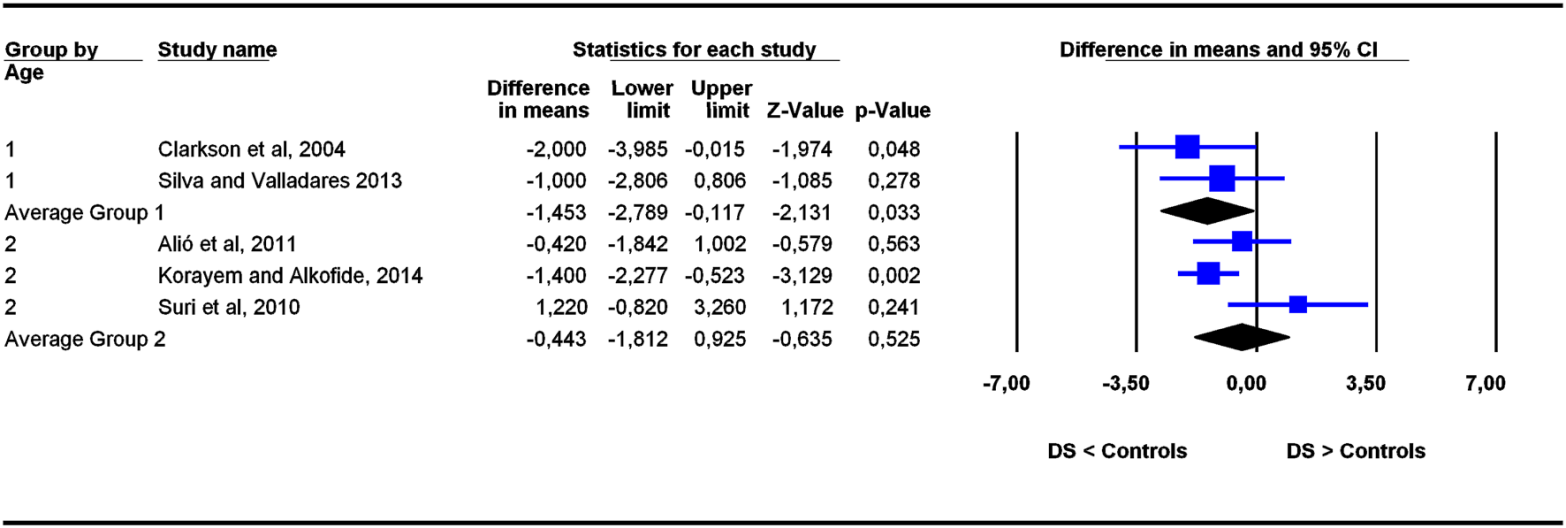
Forest plot for the outcome ‘SNA (°)’ as a function of the age group (1 = 6–11 years old), 2 = 5–22 years old).

**Figure 8:**
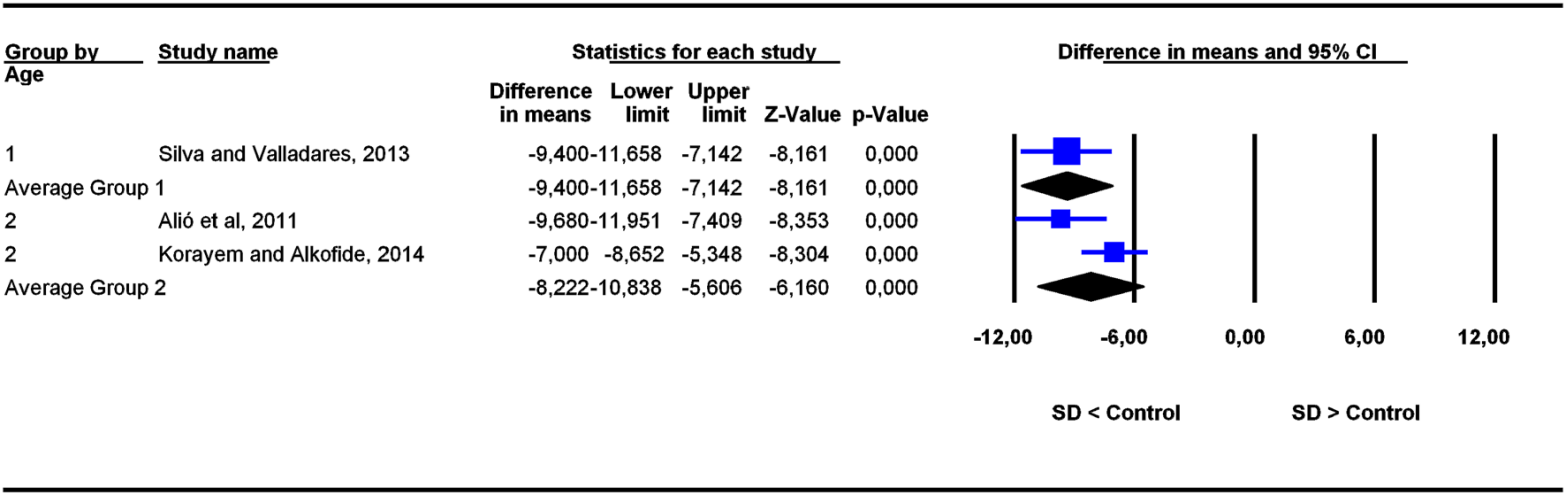
Forest plot for the outcome ‘CoA (mm)’ as a function of the age group (1 = 6–11 years old), 2 = 5–22 years old).

**Figure 9:**
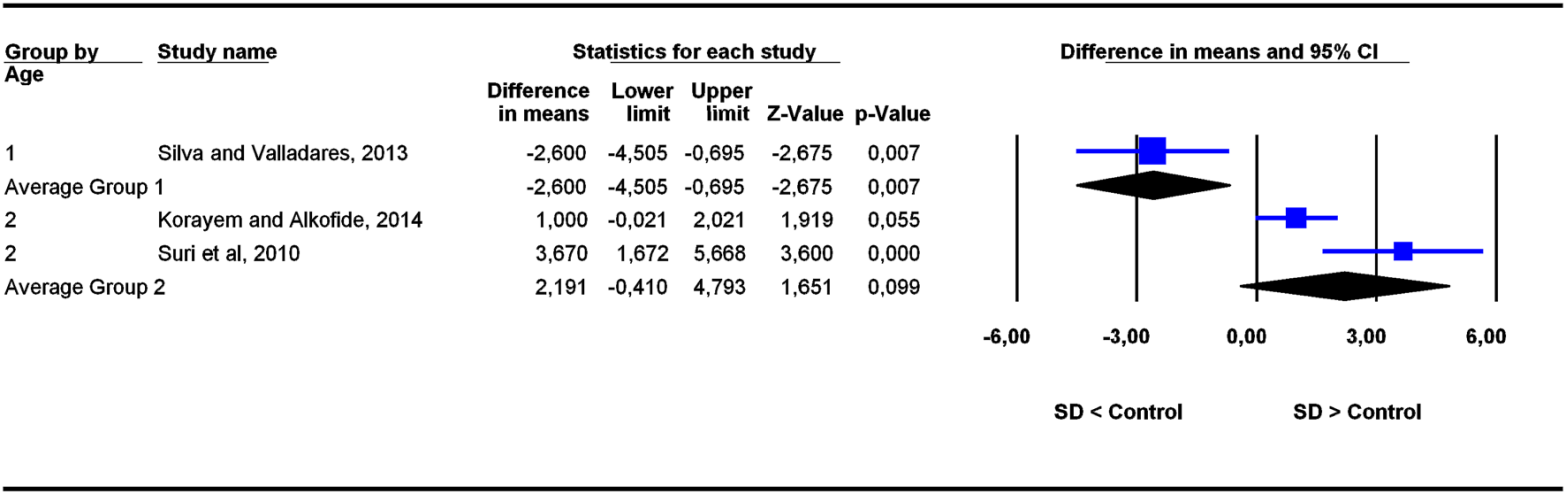
Forest plot for the outcome ‘SNB (°)’ as a function of the age group (1 = 6–11 years old), 2 = 5–22 years old).

**Figure 10:**
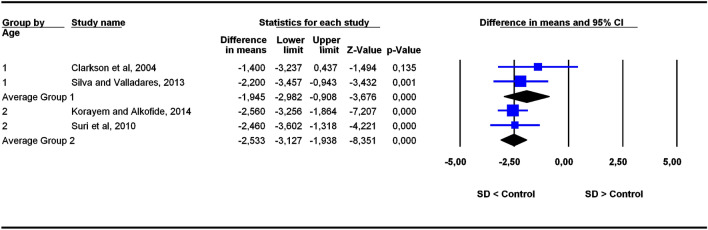
Forest plot for the outcome ‘ANB (°)’ as a function of the age group (1 = 6–11 years old), 2 = 5–22 years old).

**Figure 11:**
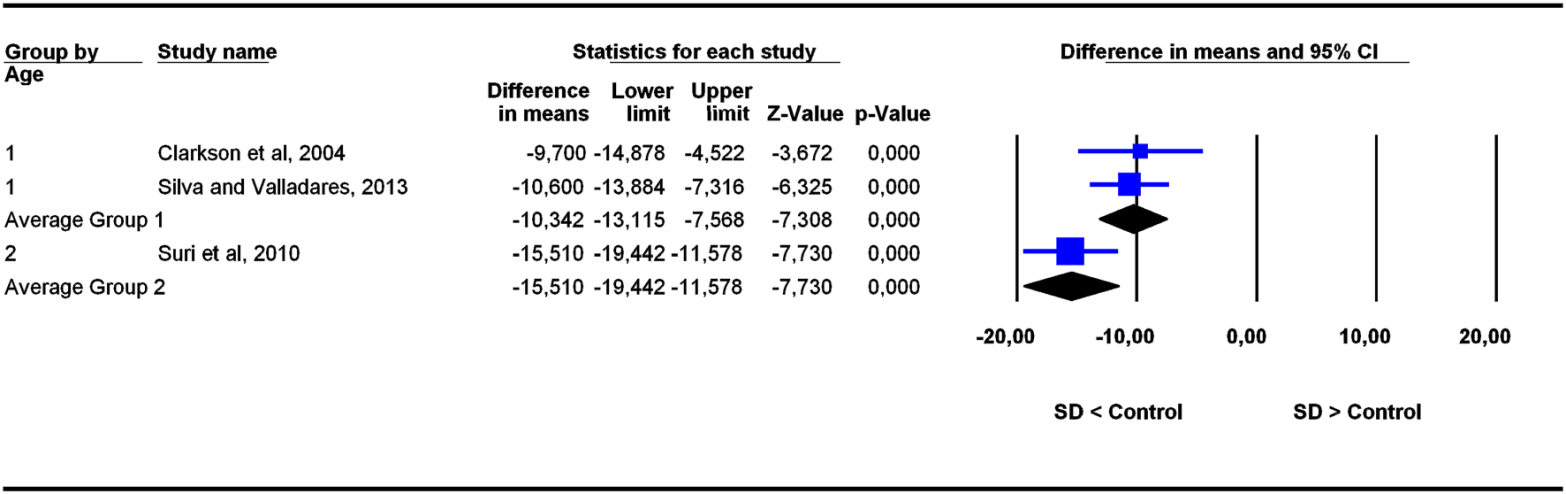
Forest plot for the outcome ‘NMe (mm)’ as a function of the age group (1 = 6–11 years old), 2 = 5–22 years old).

**Figure 12:**
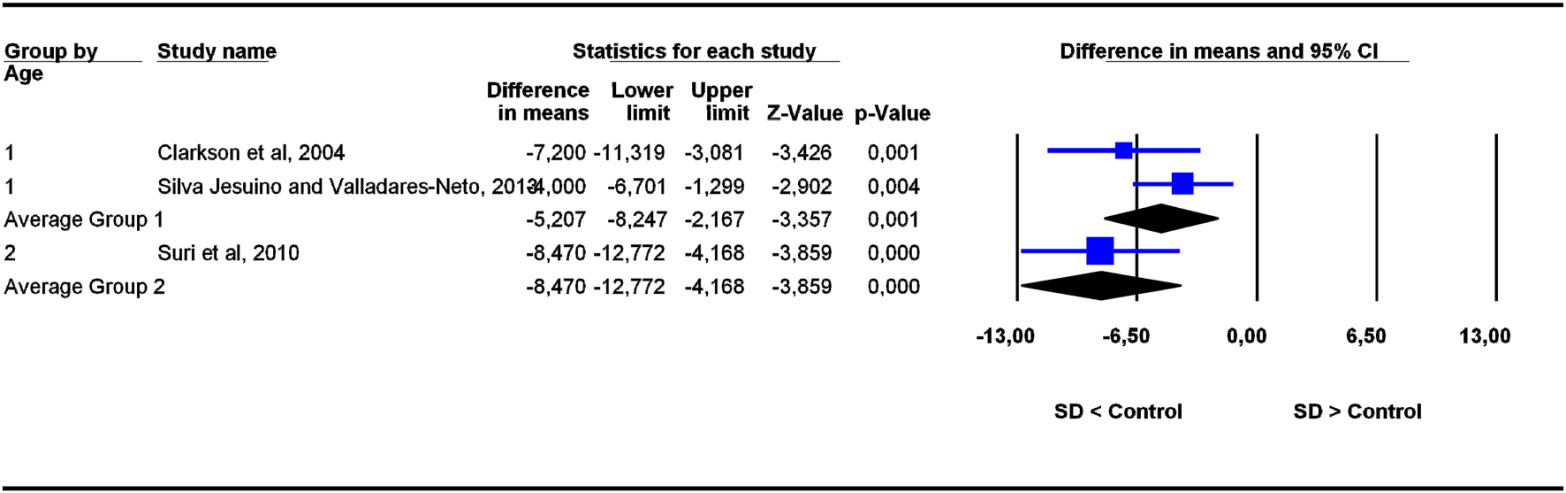
Forest plot for the outcome ‘SGo (mm)’ as a function of the age group (1 = 6–11 years old), 2 = 5–22 years old).

The studies were grouped in two categories depending on participants’ age ranges (6–11 and 5–22 years old), the former category being more homogeneous than the latter. To assess the potential influence of age ranges on effect sizes, subgroup analyses were carried out for each outcome. Forest plots (Figs. 3, 4, 5, 6, 7, 8, 9, 10, 11, 12) show the results. In addition, the *Q*_B_ statistic for testing statistical significance in differences between the two average effect sizes is shown in Table 5. Statistically significant differences were found between the average effect sizes of the two age range categories for outcomes SNBa (*p* = 0.039); SNB (*p* = 0.004) and NMe (*p* = 0.035). Regarding the outcome SNBa, both age ranges exhibited a larger mean effect size for DS cases than for controls, but the 5–22 year age range exhibited a larger difference (MD_+_ = 8.854) than the 6–11 year age range (MD_+_ = 6.500). For the outcome SNB, the significant difference found between average effect sizes of the two age ranges was due to a larger mean effect in the DS group than the controls for the category 5–22 years (MD_+_ = 2.191), whereas the inverse was found for the category 6–11 years (MD_+_ = −2.600). Lastly, for the outcome NMe, lower mean values were found in the DS group than the controls for both age ranges, but the average mean difference was more pronounced in the category 5–22 years (MD_+_ =  −15.510) than 6–11 years (MD_+_ =  −10.342).

**Table 5:** Results of the subgroup analyses for the two categories of age ranges.

Outcome	*Q*_B_	*df*	*p*
SN (mm)	2.01	1	.156
SBa (mm)	1.41	1	.235
NBa (mm)	2.52	1	.112
SNBa (°)	4.24	1	.039
SNA (°)	1.07	1	.301
CoA (mm)	0.45	1	.504
SNB (°)	8.48	1	.004
ANB (°)	0.93	1	.335
NMe (mm)	4.43	1	.035
SGo (mm)	1.47	1	.225

*Q*_B_, statistic for testing the statistical significance the difference between the mean effect sizes for the two age ranges. *df*, degrees of freedom of the *Q*_B_ statistic. *p*, probability level of the *Q*_B_ statistic.

## Discussion

The aim of this meta-analysis was to determine whether the craniofacial cephalometric characteristics of individuals with DS differ from those of the general population.

It was decided not to limit the literature search by language or date of publication in order not to miss any article that might provide information relevant to the work’s hypothesis; as a result, articles in three languages were included.

When evaluating the comparability of the cases and controls by means of the NOS, the age and ethnicity of subjects were considered, as cephalometric measurements may be influenced by these variables. Although in the seven works included for meta-analysis, age ranges and ethnicity were the same in case groups and control groups, there was some variability between the articles, especially regarding age, so in order to maintain homogeneity, the works were grouped according to age ranges into two groups: 6–11 years^27,28^ and 5–22 years^22–26^.

The seven studies included in meta-analysis used different cephalometric measurements, and so it was decided to analyze only those measurements that were repeated in at least three articles. Nevertheless, the maximum number of works to coincide in any single measurement was five. For this reason, the results of the present meta-analysis should be interpreted with caution.

Cranial base length was evaluated by means of the measurements SN, BaS and BaN. It was found that anterior cranial base length (SN) was significantly shorter in DS subjects than among control subjects. Some authors have suggested that the lesser development of the anterior cranial base is related with smaller brain size^27^. But according to Enlow the growth of the cranial base is considered rather autonomous, as compared to the cranial vault, because of that, we think that the smaller brain size of DS individuals should not be considered the main cause or the only cause of the short anterior cranial base of these patients. Rather both facts could be the effect of a different primary cause^30^. One study evaluated cranial base growth in subjects aged 8–18 years, observing that it grew in the same proportion among DS individuals as control subjects, showing that the deficit in size was produced before the age of 8 years^22^. Some authors have suggested that the deficit is of prenatal origin^9^, while others state that is develops during the first 4 years of life^31^.

As for posterior cranial base (BaS), it was also found to be significantly reduced in size in DS groups. A histological study based on autopsies of individuals at different ages determined that growth in this region comes to an end at the age of 18 years in healthy individuals^32^. Alió et al. observed that, in individuals with DS, the rate of growth decreases gradually up to the age of 15 years, while in control subjects it continues to grow, so that spheno-occipital synchondrosis growth stops earlier in individuals with DS^22^.

Obviously, the reduced anterior and posterior cranial bases in cases of DS mean that the total cranial base length (BaN) is also significantly smaller than in the general population.

At the same time, the cranial base angle (SNBa) was significantly larger in DS than control subjects. It has been suggested that delay in intra-sphenoidal synchondrosis fusion in postnatal life is essential to the cranial base’s flattening mechanism. Radiographic data obtained from individuals with DS show a delay in fusion, so that synchondrosis remains without fusing between the ages of 1–7 years, while in the general population it is fully obliterated by the end of the first year of life^33^. The present findings show that the difference in measurement between DS cases and control subjects was more acute in the 5–22 year age range. But the difference in effect size between the 6–11 year and the 5–22 year age group was 2.35°, so even though this difference was statistically significant, it was not clinically significant, as the standard deviation for this angle is around 4°^34^. In addition, according to Bjork, the cranial base angle is 130.8° ± 4.2° at age 12 and 131.6° ± 4.5° at age 20 years, showing an insignificant change from the former age to the latter^34^. Almeida et al., in their systematic review showed that SNBa remains constant from 5 to 15 years of age^35^. Alió et al. observed that in both individuals with DS and the general population it does not vary between the ages of 8–18 years^22^.

The anteroposterior position of the maxilla was evaluated by means of the SNA angle, which, although smaller in cases of DS, did not show significant differences in comparison with control subjects. The SNA angle relates the maxilla to the cranial base, which, as stated above, is significantly shorter in individuals with DS. Anteroposterior and vertical cephalometric measurements for the maxilla and mandible based on an anomalous cranial base can lead to erroneous cephalometric interpretation. For this reason, Jesuino and Valladares suggest that in these cases cephalometric measurements should take the Frankfort plane as reference^27^. The reduced anterior cranial base in DS can make the SNA similar to that of control subjects due to a geometric effect, even though the maxilla is smaller^23^, because the N point will be located at a more posterior location^24^.

Maxillary length (CoA) was significantly smaller in DS than control groups, corroborating the existence of maxillary hypoplasia in the sagittal plane. Alió et al. showed that the maxilla grows in the same proportion between the ages of 8 and 18 years in DS cases as in healthy subjects^24^. Klingel et al. observed that at the age of 6–9 months the maxilla is already smaller in all three dimensions in DS infants^36^.

As for the mandible, no statistically significant differences were observed between DS and control subjects in SNB angle, although values were higher in DS subjects. This measurement is also related to the cranial base; in this case shortening produces larger SNB angles^23^. At the same time, the present work found that the cranial base angle was significantly larger in DS groups, which would lead to less symphysis projection, while in control subjects the angle was more acute, contributing to a more anterior mandibular position^35^. Again, it is clear that to assess cases of DS, it is necessary to use a reference plane other than the cranial base to determine the anteroposterior position of the mandible correctly, and to reach clear conclusions regarding the size of the mandible. Meanwhile, in the 6–11 year age range, SNB angle values were lower in DS subjects than controls, while in the 5–22 year range it was larger in DS groups. This could be a result of the fact that the mandible has not undergone full growth in the younger age group. Mandibular growth ends around the age of 17 in females and 19 in males^37^.

Relating maxillary and mandibular anteroposterior position by means of the ANB angle, it was found that this was significantly smaller among DS subjects, indicating a greater tendency among these individuals to present skeletal Class III malocclusions.

Both anterior (NMe) and posterior face height (SGo) were significantly smaller among individuals with DS. In these cases, maxillary hypoplasia in the vertical plane conditions developmental deficiency in the facial middle third^24^. For NMe, the difference between DS groups and control subjects was more pronounced in the 5–22 than the 6–11 age range, probably due to the fact that the latter group had still not undergone complete growth.

The tendency toward brachyfacial pattern in individuals with DS was notable, in spite of the muscular hypotonia that such cases usually present, the tendency to keep the mouth half open in repose, and frequent infection of the upper airway. This is perhaps due to mandibular autorotation in maximum intercuspation resulting from maxillary vertical hypoplasia. The meta-analysis by Oliveira et al. found a higher prevalence of anterior open bite among individuals with DS, although they reported a low level of evidence for an association between DS and anterior open bite^38^.

Although the present work suffered several limitations, it was possible to reject the hypothesis that there would be no differences in craniofacial characteristics between individuals with DS and a healthy population, as clear differences were found. Nevertheless, in order to reach clear conclusions it would be necessary to unify the cephalometric measurements taken in different studies, and to use different reference planes for SN in order to determine vertical and anteroposterior positions of the maxilla and mandible. The results must be interpreted with great caution due to the scarce number of studies that reported each outcome measure. In addition, the small number of studies included in meta-analysis did not make it possible to examine the potential effects of publication bias, as at least 10 studies are needed to apply the typical methods for assessing publication bias.

## Conclusions

In spite of the limitations of the present meta-analysis, it may be concluded that individuals with DS present a cranial base of reduced length, which is more flattened than in healthy subjects. Moreover, the maxilla is of reduced size in the sagittal plane and there is a tendency toward skeletal Class III malocclusion. DS is also characterized by reduced facial height in both posterior and anterior regions.
